# UV-Synthesized Polyacrylamide-Based Polymer Sensor for Measuring Soil–Water Characteristic Curves in Unsaturated Soils

**DOI:** 10.3390/polym18141692

**Published:** 2026-07-09

**Authors:** Anar Arinova, Alfrendo Satyanaga, Gulnur Kalimuldina, Rezat Abishev, Eriko Dewangga, Saltanat Orazayeva, Jong Kim

**Affiliations:** 1 Department of Civil and Environmental Engineering, School of Engineering and Digital Sciences, Nazarbayev University, Astana 010000, Kazakhstan; anar.arinova@nu.edu.kz (A.A.); rezat.abishev@nu.edu.kz (R.A.); eriko.dewangga@nu.edu.kz (E.D.); saltanat.orazayeva@nu.edu.kz (S.O.); jong.kim@nu.edu.kz (J.K.); 2 Department of Mechanical and Aerospace Engineering, School of Engineering and Digital Sciences, Nazarbayev University, Astana 010000, Kazakhstan; gkalimuldina@nu.edu.kz

**Keywords:** soil–water characteristic curve, soil suction, polymer sensor, polyacrylamide, unsaturated soils

## Abstract

This study presents the development and evaluation of a hydrogel-based superabsorbent polymer sensor (HSPS) for measuring soil suction and establishing the soil–water characteristic curve (SWCC) of unsaturated soils. Polyacrylamide (PAM) hydrogels were synthesized via UV-induced free radical polymerization using acrylamide with varying crosslinking degrees. The polymers were characterized through FT-IR and TGA analyses, confirming successful synthesis and high thermal stability. Swelling, water retention, and kinetic behavior were systematically investigated. Results indicated that lower crosslinking density significantly enhanced swelling capacity, reaching up to 3000% in distilled water, while saline environments reduced absorption due to ionic screening effects. Swelling kinetics followed anomalous (non-Fickian) diffusion behavior and were well described by the pseudo-second-order Schott model. The synthesized polymers were integrated into a modified high-sensitivity pressure sensor operating on the osmotic principle to measure matric suction. The system was validated using natural soil. Among the tested formulations, the HSPS-3 demonstrated the most reliable suction measurements, reaching values up to approximately 1 MPa without significant temperature sensitivity. The resulting SWCC exhibited bimodal characteristics consistent with the soil’s dual pore structure. The proposed method provides a cost-effective, simple, and efficient alternative for suction measurement, expanding the practical range of SWCC determination in unsaturated soil mechanics.

## 1. Introduction

Polymer materials with high swelling capacity, commonly referred to as hydrogel-based superabsorbent polymers (HSPs), have attracted increasing attention due to their ability to absorb and retain large amounts of water relative to their dry mass. Superabsorbent polymers (SAPs) represent a class of hydrophilic polymer networks, characterized by a three-dimensional structure capable of absorbing and retaining water hundreds of times greater than their own weight [1,2,3]. These three-dimensional polymeric networks do not dissolve in water or aqueous solutions due to the presence of chemical or physical crosslinks, which provide structural stability while allowing extensive swelling [4]. Recently, the design and development of HSPs has attracted widespread attention owing to their remarkable swelling, which has enabled broad applications in agriculture [5], forestry [6], health care, geotechnical engineering [7,8], and functional sensors [9,10]. Polymer sensors using HSPs provide a sensitive and efficient platform for evaluating water absorption and retention dynamics in porous media like soils, enabling significant analysis of soil–water interactions, especially in studies of unsaturated soil properties.

Acrylamide (AM) has become a preferred monomer for the synthesis of superabsorbent polymer owing to its low production cost, wide availability, and favorable processing characteristics. The high hydrophilicity of AM facilitates the development of PAM hydrogels [11], whose hydrophilic functional groups (–NH_2_ and –OH) make them highly effective as water-absorbing agents in various applications. In addition, AM is characterized by high reactivity, exhibiting one of the highest k_p_/k_t_ ratios among known monomers, where k_p_ and k_t_ represent the rate constants of propagation and termination in radical polymerization, respectively. This unique reactivity results in the formation of polymers with exceptionally high molecular weight [12]. UV irradiation polymerization simplifies PAM synthesis, a widely studied SAP, yielding high-molecular-weight polymers with excellent structural stability. Compared to traditional methods, UV-assisted synthesis provides a faster, cleaner, and more energy-efficient hydrogel fabrication process.

In geotechnical engineering, one of the most fundamental relationships governing the hydraulic and mechanical behavior of unsaturated soil is described by SWCC. SWCC defines the correlation between matric suction and volumetric water content, thereby providing crucial insights into permeability, shear strength, and compressibility of soil [7,13]. Conventional laboratory methods for determining SWCC are labor-intensive, time-consuming, and require sophisticated equipment, which limits their applicability for large-scale and rapid testing.

Over the years, significant advancements in technology have improved the accuracy of SWCC measurements. One of the earliest tools developed for this purpose is the tensiometer, which functions based on capillary action. As noted by [14], the field-application tensiometer was first introduced in the early 1920s by Lorenzo A. Richards. Standard tensiometers are limited to measuring soil suction up to 100 kPa due to cavitation occurring in the water column or the ceramic cup. Despite this limitation, tensiometers remain widely used for field measurements due to their relatively straightforward operation.

To overcome the low-capacity constraint, Ridley and Burland [15] designed the high-capacity tensiometer (HCT), capable of recording suctions up to 1500 kPa. However, HCT readings are significantly affected by ambient temperature variations, necessitating temperature calibration prior to use [16,17,18]. Another method, the dew-point technique, utilizes a chilled mirror to determine water potential. This method becomes effective only when vapor equilibrium is established, making it suitable for measuring high suction values typically above 1 MPa and up to 300 MPa [19,20]. These techniques generally rely either on capillarity (due to water’s surface tension) or on estimating suction via the relative humidity around the soil sample. An alternative approach employs the osmotic principle, which is governed by the movement of water across a semipermeable membrane due to solute concentration differences—commonly using polymers as the osmotic agent.

Lagerwerff et al. [21] were the first to utilize polymers in a plant culture setting. Later, Peck and Rabbidge [22,23] modified this technique as an osmotic tensiometer used in agricultural applications. Kassiff and Shalom [24] have widened its application into geotechnical engineering by introducing an osmotic oedometer. Inspired by these studies, Bocking and Fredlund [25] developed an osmotic tensiometer for geotechnical applications but faced the difficulties of chamber leakages due to compaction in materials and sealing techniques available at that time. Ever since, a few impressive improvements in the development of osmotic tensiometers have been observed. Researchers, including Biesheuvel et al. [26,27], Bakker et al. [28], Van der Ploeg [29], Van der Ploeg et al. [30], Liu et al. [31,32,33], and Hamdany et al. [8], presented different implementations, including the use of diverse polymers and the ability to withstand temperature changes. Osmotic methods usually require a lower workload for measurements when capillary methods are used as the reference techniques.

Despite significant advances in osmotic tensiometers and hydrogel-based suction measurement systems, insufficient attention has been devoted to investigating the influence of the crosslinking density of polyacrylamide hydrogels on their suitability as sensing media for soil suction measurements. Most previous studies have primarily focused on sensor design and measurement methodologies, whereas the relationship between hydrogel swelling behavior, water retention properties, and soil suction sensing performance remains inadequately understood. Therefore, the present study investigates a series of UV-synthesized polyacrylamide hydrogels with different crosslinking densities and systematically evaluates their physicochemical characteristics, swelling behavior, and performance in an HSPS system for soil suction measurements.

The main output of this research is the establishment of the SWCC using the UV-synthesized HSPS, which can extend the measurement of suction up to 1000 kPa with long-term durable performance. The scope of this work includes a comprehensive literature review on polymers and SWCC measurement techniques, polymer synthesis and characterization (including swelling behavior and Fourier Transform Infrared Spectroscopy (FT-IR) analysis), index property assessment, unsaturated soil testing, and the derivation of the SWCC.

## 2. Materials and Methods

### 2.1. Materials

Acrylamide (AM, C_3_H_5_NO, Sigma-Aldrich, St. Louis, MO, USA, ≥99%), N, N′-methylenebisacrylamide (MBA, (CH_2_=CHCONH)_2_CH_2_, Sigma-Aldrich, ≥99.5%), 2-hydroxy,2-methylpro-piophenone (HMP, HOCH_2_CH_2_OC_6_H_4_COC(CH_3_)_2_OH, Sigma-Aldrich, 98%), sodium chloride (NaCl, Sigma-Aldrich, ≥99%), and calcium chloride (CaCl_2_, anhydrous, Sigma-Aldrich, ≥97%). All the experiments included deionized water.

### 2.2. Materials Characterization

The chemical structure of the samples was analyzed using Fourier Transform Infrared Spectroscopy (FT-IR, Nicolet iS10 FT-IR Spectrometer, Thermo Fisher Scientific Inc., Waltham, MA, USA). Additionally, the thermal stability and compositional properties of polyacrylamide were evaluated through Thermogravimetric Analysis (TGA, STA 6000, PerkinElmer, Shelton, CT, USA) at a heating rate of 10 °C/min from 25 to 800 °C under an inert nitrogen atmosphere.

### 2.3. Synthesis

**Figure 1:**
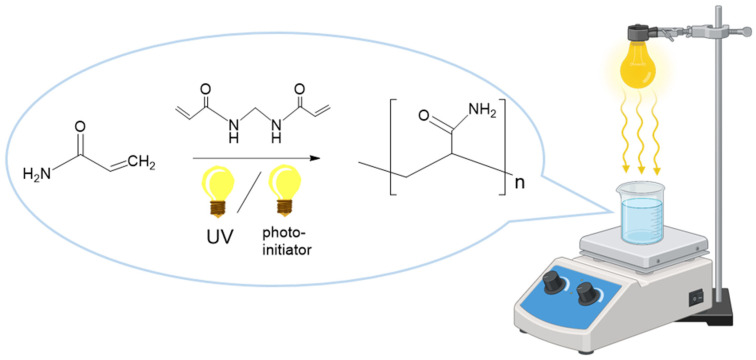
Schematic illustration of UV polymerization of PAM.

PAM was synthesized by utilizing the UV polymerization method, enabling the formation of the polymer with a high molecular weight. The UV radiation source used in this study had a wavelength range of 345 to 385 nm. The chemicals used for the synthesis include AM as the monomer, MBA as the crosslinking agent, and HMP as the photoinitiator. For the synthesis of PAM, a predefined amount of AM and MBA was initially dissolved in distilled water in a beaker (Table 1). Subsequently, a trace amount of HMP was dissolved in the solution, followed by deaeration in bubbling nitrogen for 10 min. The mixed solution was exposed to the UV light source for 1 h to complete the polymerization. After the polymer’s extraction from the beaker, the hydrogel was cut into small pieces. After that, they were immersed in distilled water at room temperature for 1 week, with the daily water changes to eliminate unreacted components, and dried at 80 °C in a vacuum oven for 48 h (Figure 1).

### 2.4. Water Absorption Test

The swelling behavior of samples was analyzed by rehydrating and measuring the change in weight as a function of dry weight. The weight after UV-polymerization was recorded before placing each sample in 40 mL of distilled water. Once equilibrium swelling was reached in the samples at 25 °C (time 24 h to 120 h), swollen weight ($W_{s}$) was recorded. The percentage swelling of the samples was calculated using Equation (1) [34]:

(1) $$Swelling\;\left(\%\right)=\left(\frac{W_{s}-W_{d}}{W_{d}}\right)\times 100,$$

where $W_{s}$ and $W_{d}$ refer to the samples in the equilibrium swollen state and dried state, respectively.

### 2.5. Water Retention Test

After the samples reached their equilibrium state of water absorption, the water retention test was carried out. The swollen samples were kept under ambient laboratory conditions, 24–25 °C and approximately 25% relative humidity, and weighed at predetermined time intervals over a period of 120 h. The water retention rate (*WRR*) at a given time was calculated using Equation (2) [35]:

(2) $$WRR\left(\%\right)=\left(\frac{W_{t}-W_{d}}{W_{o}-W_{d}}\right)\times 100,$$

where $W_{t}$ corresponds to the mass of the swollen hydrogel at time *t*, $W_{0}$ is the initial mass of the swelling, and $W_{d}$ refers to the mass of the dried sample.

### 2.6. Water Absorbency Kinetics

The kinetics of water absorption were determined by soaking 0.1 g of sample in excess distilled water and calculating the amount of *Q*. Kinetic data were recorded at 1 h intervals and analyzed using the Fick diffusion (3, 4) model and the Schott second-order kinetic model (5).

(3) lnF=lnk+nlnt,

(4) $$F=\frac{Q_{t}}{Q_{w}},$$

(5) $$\frac{t}{Q_{t}}=\frac{l}{kQ_{w}^{2}}+\frac{t}{Qw},$$

where $F=Q_{t}/Q_{w}$—the swelling ratio at time t, $Q_{t}$—the sample mass at time t, $Q_{w}$—the equilibrium mass, k—the kinetic constant, n—the diffusion exponent characterizing the water transport mechanism, t—the time.

### 2.7. Preparation of Polymer Sensor

The HSPS sensor (KELLER development for 35X series) was originally developed as a pressure transducer, equipped with an electrically isolated piezoresistive N-type sensor laser-trimmed in a chamber located below the design and suitable for many applications. First, the sensor could measure only positive pore-water pressure, and not negative pore-water pressure (soil suction). KELLER [36] reported that the pressure transducer can measure over a range of 3 MPa, with an error lower than 0.05%, and operates well within a temperature range from −40 °C to 125 °C. These features made the sensor especially fit for measurements at matric potential beyond the wilting point. A new cover was next introduced to the sensor, thus allowing the pressure transducer to operate as a conventional tensiometer.

**Figure 2:**
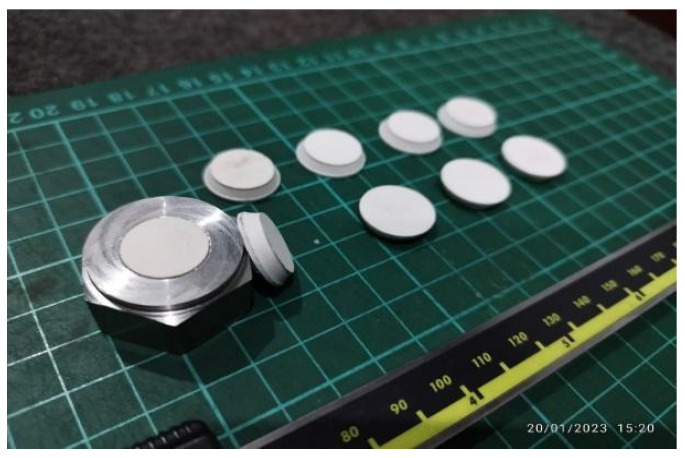
The manufacture of the modified cap.

**Figure 3:**
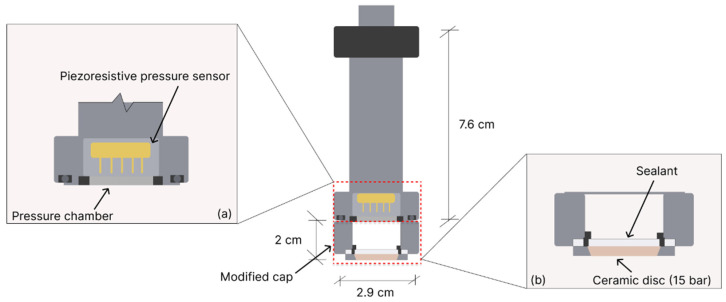
Cross-section of HSPS: (**a**) piezoresistive sensor and pressure chamber; (**b**) ceramic disc attached to modified cap.

The modified cylindrical cap is a steel solid with improved strength and durability, containing a hollow chamber based on the Tempe Cell ceramic disc and A_15-30 bar (155 mm) with 15.7 mm diameter at its center section, as shown in Figure 2 and Figure 3. Silicon has been used as an air-sealing material surrounding the ceramic disc to prevent air flow and leakage through the chamber. The performance of the HSPS has been tested by Liu et al. [31,32,33] and Hamdany et al. [8]. Aventian et al. [37,38] applied HSPS with polyacrylamide, a widely accepted polymer, to determine soil suctions. Experimental results are given for comparisons between soil suctions measured within the HSPS and those of one chosen Tempe Cell (axis-translation), showing that such measurements can be done. The transference of HSPS to the pressure plate (up to 1500 kPa) also needs more research. At present, the HSPS does not need performance improvements with respect to the device, but rather in the polymer that is used as a measuring medium for soil suction in this tool.

The HSPS was linked with a K-114B interface using RS485 for computer control. This RS485 interface connects the K-114B to both a common micro-USB and a USB-A. Before one can use the HSPS, one needs to install the KELLER CCS30 app. Prior to program execution, the polymer needs to be loaded with some care in the chamber, and each properly adapted cap must be placed. The operation of the HSPS depends upon the ability of the polymer to swell/shrink with changes in the osmotic gradient between free water and the polymer. When swelling and imbibition occur, acting against the chamber from all sides is the polymer-imbiber complex, which generates pressure on a piezoresistive sensor to measure negative pressure under controlled conditions. Inversely, contraction of the polymer causes a similar response and reduction in pressure.

Saturation of the HSPS should come before soil suction monitoring. The saturation of the HSPS in the polymer was achieved via immersion in distilled water. Distilled water was selected for use as it does not contain unwanted minerals or salts, and its presence in the solution has been shown to affect the soil suction generated by the polymer [27]. The saturation period lasted for about 72 h in order to reach an equilibrium status and also to minimize a possible long-term degradation of the fully saturated state.

The HSPS is used to quantify a higher soil suction using a polymer enclosed within the sensor chamber. Upon saturation, water enters the chamber through the ceramic disk and is absorbed by the polymer, causing it to swell until maximum swelling is reached. Soil suction is determined based on the inverse relationship between the pressure acting on the diaphragm and the soil osmotic suction. Aventian et al. [38] stated that increased pressure corresponds to decreased soil suction, and vice versa. The swollen polymer exerts pressure on the piezoresistive sensor located beneath the pressure chamber, producing the maximum pressure reading. When the HSPS is placed in contact with the soil, the osmotic imbalance causes water to leave the polymer, resulting in polymer contraction. Consequently, the pressure acting on the diaphragm decreases compared with its fully saturated condition. Based on this inverse relationship, soil suction is determined from the difference between the maximum pressure acting on the diaphragm under saturated conditions and the instantaneous pressure measured when the HSPS is in contact with the soil. Following Aventian et al. [38], the soil suction was calculated using Equation (6):

(6) $$\;\;\psi =p_{s}-p_{ij},$$

where $p_{s}$ is the maximum pressure acting on the diaphragm under saturated conditions (kPa), ψ is the suction of soil (kPa), and $p_{ij}$ is the instantaneous pressure acting on the diaphragm during measurement (kPa).

### 2.8. Soil Properties

**Figure 4:**
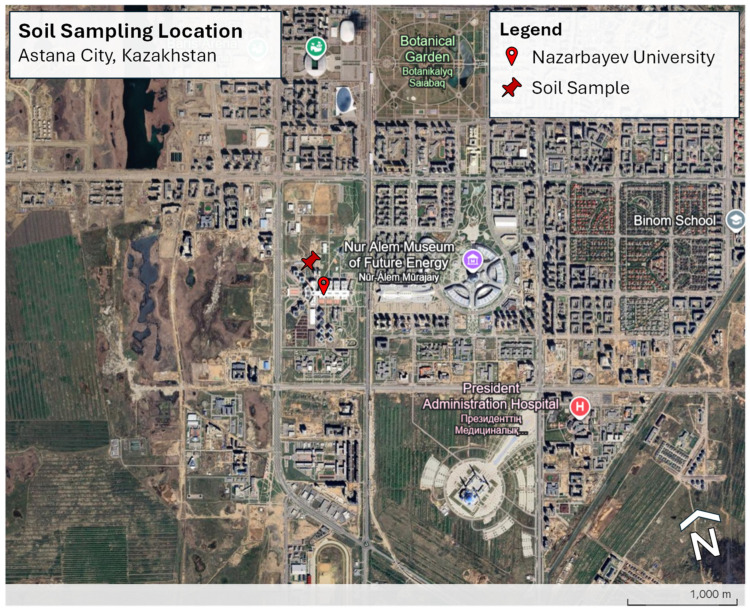
Location of soil sampling (extracted from Google Earth, 2024).

**Figure 5:**
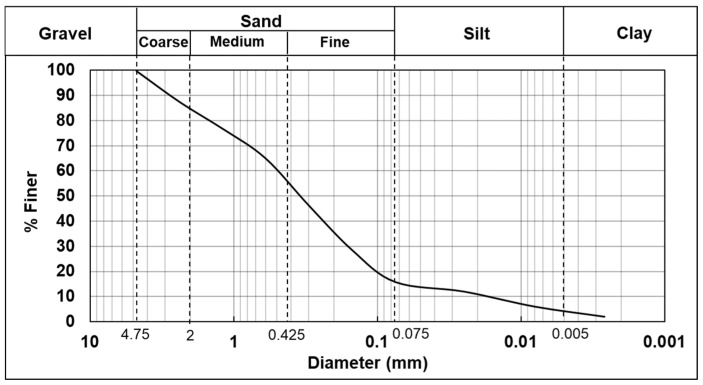
Grain size distribution.

The soil used in the present study is from Astana, Kazakhstan. Specifically, the soil was harvested from Turan Avenue during the study period in Nazarbayev University (called Turan’s soil) as presented in Figure 4. Sampling was based on disturbed soil to a depth of 2 m. ASTM test methods for index properties of the soil used are shown in Table 2. The grain-size distribution curve is given in Figure 5.

**Figure 6:**
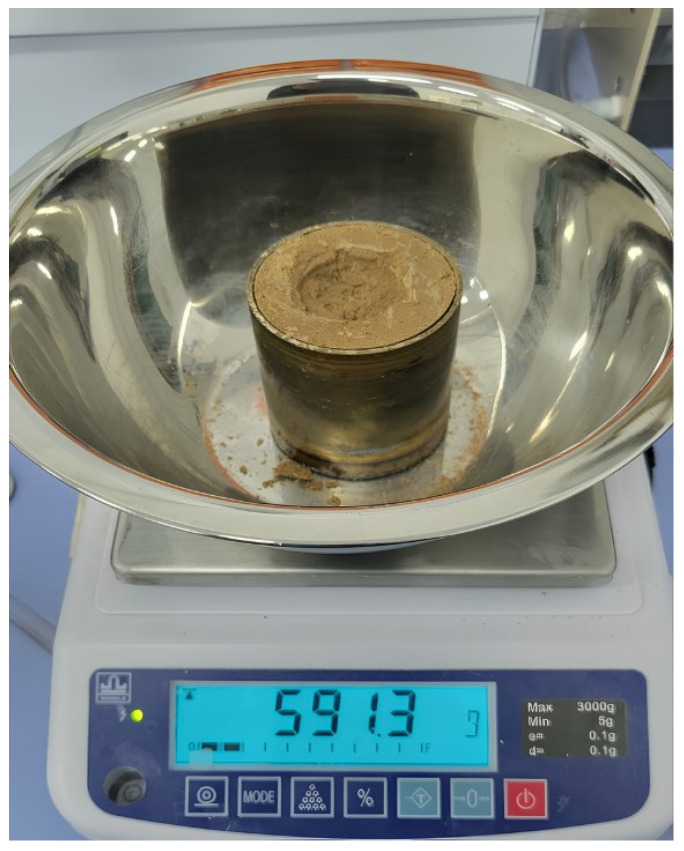
Preparation of soil specimen for SWCC test using HSPS.

Prior to testing, the soil was compacted using a ring at under Maximum Dry Density (MDD), and both the surfaces of the samples were slightly excavated to match their diameters with those of HSPS. This pre-treatment allowed for the soil to have an improved reaction with the apparatus (Figure 6). Subsequently, the sample was saturated.

### 2.9. Soil–Water Characteristic Curve (SWCC)

**Figure 7:**
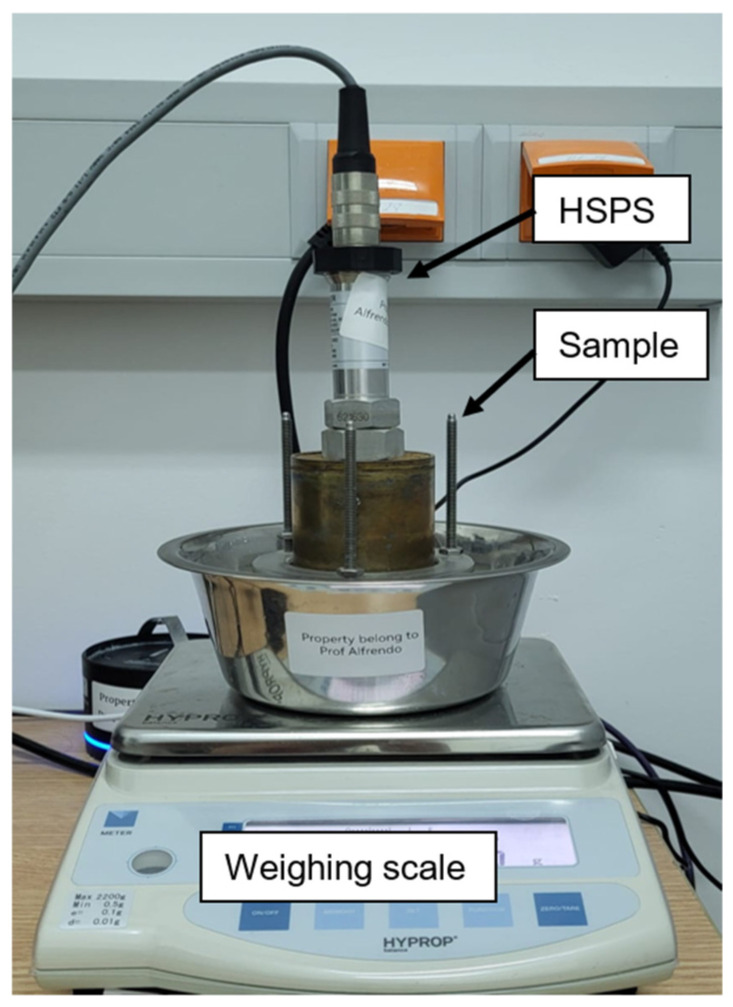
SWCC monitoring setup.

The specimen was compacted at MDD. Its external surface was also shaped according to the radius of the HSPS (Figure 5) and pre-moistened for SWCC reading. Soil was saturated by means of water infiltration from the bottom up, excluding air trapped in the pores. The saturation conditioning was continued for 1 week until the samples’ weight became constant (full saturation). Then, the SWCC under different polymers was determined based on their effect on soil suction measurements. The readings were collected by using a regular weighing scale, because the HSPS measures only soil suction (for monitoring water content changes over time, water content measurements are required). So, it was critically important that the HSPS be perfectly horizontal with respect to the sample and that there were no gaps between their caps and soil, or these results would become meaningless. The SWCC test was performed only in the drying part of the curve, where the sample was exposed to open-air conditions to ensure evaporation (Figure 7).

To minimize the gap between low and moderate suctions (i.e., at high RH values) compared with that between low/moderate and high suctions, the WP4C instrument was used for discrete SWCC measurements up to a suction of 1.5 MPa. The WP4C measurements can be considered compatible with the HSPS measurements over the overlapping suction range, because the total suction measured by the WP4C is approximately equal to the matric suction when the osmotic suction is negligible. This is due to the non-saline nature of the tested soil, which has negligible soluble salt content. Under these conditions, the osmotic suction component is expected to be very small compared with the matric suction. The WP4C measurements were performed following the procedure described by Satyanaga et al. [19].

Data analysis was performed after completion of the SWCC monitoring. The gravimetric water content was converted to volumetric water content by multiplying it by the dry density of the soil. The measured SWCC data were then fitted using the bimodal model (Equation (7)). The soil investigated in this study is considered to have characteristics similar to those reported by Aventian et al. [38]. Soils of this kind are considered to be bimodal—that is to say, both coarse (macro-pores) and fine-grained soils (micro-pores) can affect the shape of SWCC, as a result, we get two separate sigmoidal curves. In addition, as with conventional SWCC models, the correction factor (Equation (8)) was applied to ensure zero water content at a suction of 10^6^ kPa in accordance with the laws of thermodynamics [45].

(7) $$\theta_{w}=C_{\left(\psi \right)}\left[\theta_{r}+\left(\theta_{s1}-\theta_{s2}\right)\left(1-erfc\;\frac{ln\;\left(\frac{\psi_{a1}-\psi }{\psi_{a1}-\psi_{m1}}\right)\;}{s_{1}}\right)+\left(\theta_{s2}-\theta_{r}\right)\;\left(1-erfc\;\frac{ln\;\left(\frac{\psi_{a2}-\psi }{\psi_{a2}-\psi_{m2}}\right)\;}{s_{2}}\right)\;\right],$$

(8) $$C_{\left(\psi \right)}=1-\frac{ln\;\left(1+\frac{\psi }{\psi r}\right)\;}{ln\;\left(1+\frac{10^{6}}{\psi r}\right)\;},$$

where $\theta_{s}$ is the volumetric water content at saturated condition; $\theta_{w}$ is the volumetric water content for given suction; *ψ* is the soil suction (kPa); $\psi_{a}$ is the soil air-entry value (kPa); $\psi_{m}$ is the inflection point (kPa); $\theta_{r}$ is the volumetric water content at residual condition; $C_{\left(\psi \right)}$ is the correction factor; $\psi_{r}$ is the suction at residual condition (kPa); s is the geometric standard deviation; and erfc is the complementary error function, defined as $erfc(x)=\int_{-\infty }^{x}\frac{1\;}{\sqrt{2\pi }\;}exp(-\frac{x^{2}}{2})dx$. Subscripts 1 and 2 refer to subcurve 1 (macro pores) and subcurve 2 (micro pores), respectively.

## 3. Results and Discussion on Polymer Testing

### 3.1. Chemical Characteristics of PAM

**Figure 8:**
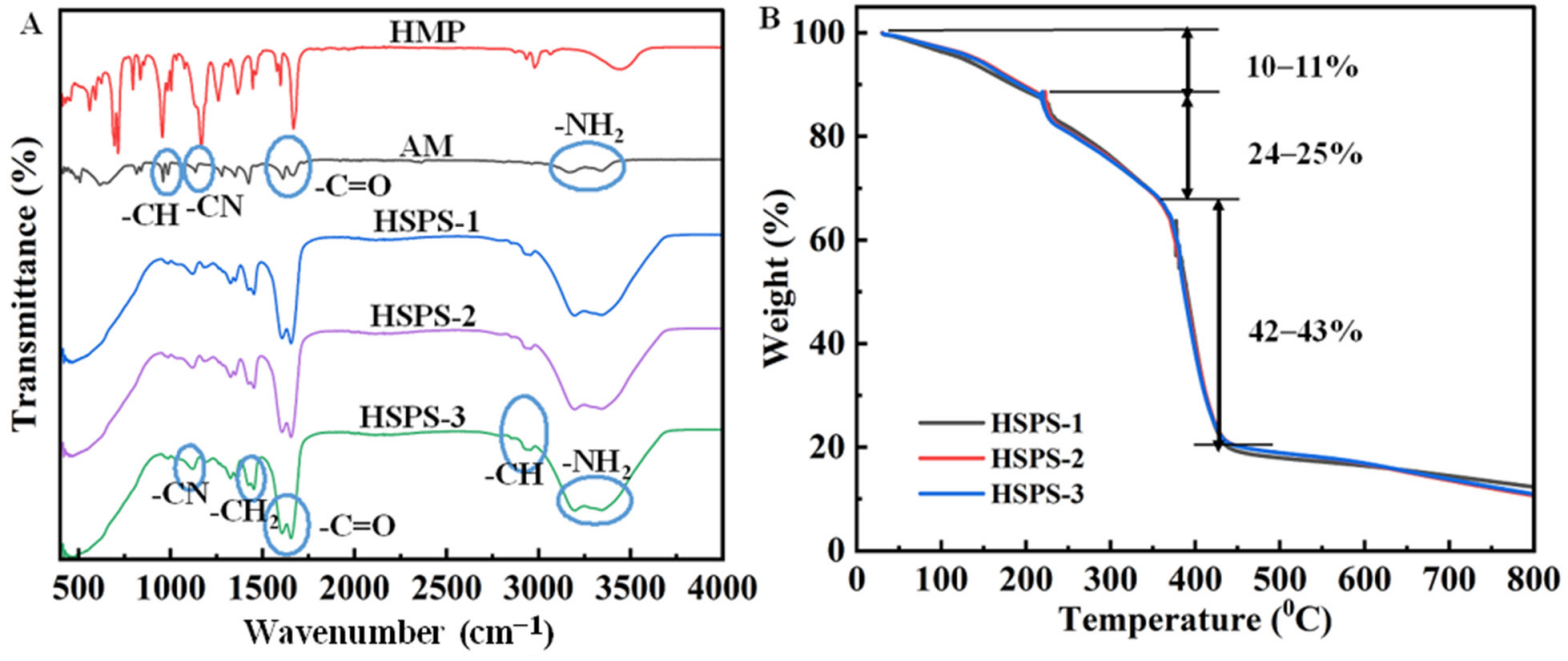
Chemical characterization of the synthesized HSPSs: (**A**) FT-IR spectra of pure AM, HMP, and synthesized HSPS-1, HSPS-2, and HSPS-3; (**B**) TGA analysis of the HSPSs.

Polymeric hydrogels PAM synthesized by free radical polymerization using UV initiation with varying crosslinking degrees were physico-chemically characterized using FT-IR and TGA. The structure of the synthesized PAM was studied using FT-IR. Figure 8A illustrates the FT-IR spectra of the synthesized PAM. The absorption peaks at 3442 cm^−1^ and 1649 cm^−1^ were attributed to the stretching vibration of amino groups (–NH_2_) and carbonyl groups (C=O) of amide in PAM. The absorption peaks at 1454 cm^−1^ were due to the C–N stretching vibration, 1319 cm^−1^ was due to the C–H bending vibration, and 1189 cm^−1^ was due to the –NH_2_ bending vibration. The absorption peak at 1432 cm^−1^ stands for =CH_2,_ respectively. In conclusion, the FT-IR spectrum showed that the chemical structure of the synthesized PAM has been confirmed [46].

TGA was characterized to evaluate the thermal stability of the prepared PAM, and the results are shown in Figure 8B. The three stages of thermal decomposition are evident here. In the first stage (100–200 °C), a 11% weight loss associated with the desorption of absorbed water was observed. In the second stage at temperatures ranging from 200 to 360 °C, weight loss of about 25% occurred, which is attributed to thermal decomposition and imidization of amide groups (–CO–NH–) [47]. In the last stage, there is a remarkable thermal decomposition at temperatures higher than 430 °C with about 43% weight loss, which may be attributed to the degradation of the PAM backbone [48]. The PAM made at 500 °C and above was completely decomposed, and its residual weight ratio was about 10%. Accordingly, the TGA analysis shows that for PAM synthesized by the UV/HMP initiation system, it has high thermal stability [49].

### 3.2. Swelling Analysis

The swelling behavior of the hydrogel is predominantly affected by surface pore size, intermolecular gaps in its three-dimensional network structure, and the chemical nature of the hydrophilic functional groups. MBA was used as a chemical crosslinking agent to form a three-dimensional polyacrylamide network during UV-induced free-radical polymerization. Crosslinking is essential because linear polyacrylamide chains are water-soluble and would dissolve in aqueous media. The formation of covalent bonds between polymer chains generates a stable hydrogel network capable of absorbing and retaining water while maintaining structural integrity. Increasing the crosslinking density reduces the network mesh size and restricts chain mobility, which generally decreases swelling capacity while improving dimensional stability and mechanical strength. Similar observations have been reported for crosslinked biopolymer systems, where crosslinking reduced the availability of hydrophilic groups and promoted the formation of a more stable three-dimensional network structure, resulting in improved durability and resistance to environmental degradation [50].

**Figure 9:**
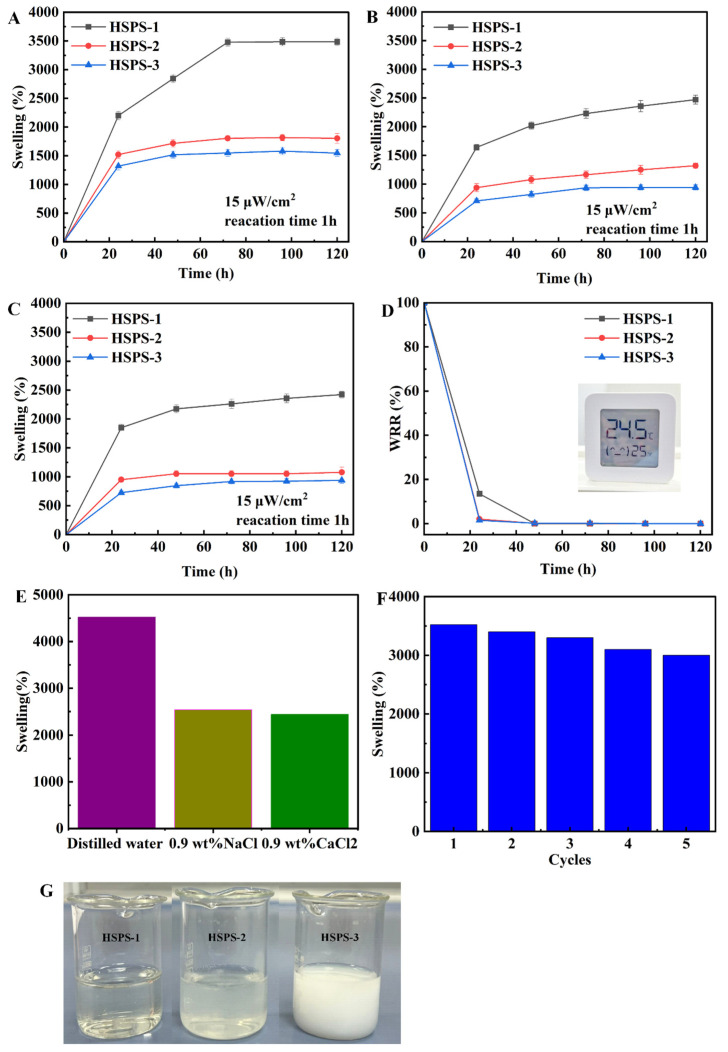
The swelling in synthesized PAM with different concentrations of the crosslinking agent: (**A**) the swelling in DI water; (**B**) the swelling in 0.9 wt% NaCl solution; (**C**) the swelling in 0.9% CaCl_2_ solution; (**D**) water retention test of PAM; (**E**) comparative diagram of PAM swelling in various solutions; (**F**) diagram consecutive swelling/deswelling cycles in DI water of HSPS-1; (**G**) visual demonstration of the degree of crosslinking of PAM. Error bars indicate ± SD obtained from three independent measurements (*n* = 3).

As shown in Figure 9A, all HSPSs exhibited two distinct swelling stages: an initial rapid swelling period during the first 20 h, followed by a slower swelling stage until equilibrium was reached. The HSPS-2 (5% crosslinking) and HSPS-3 (10% crosslinking) hydrogels approached equilibrium more rapidly than the HSPS-1 (1% crosslinking) due to their denser network structures. During the immersion test of 120 h, the samples remained intact and stable without any disintegration. The results clearly show that lower crosslinking density led to higher swelling ratios. The HSPS-1 exhibited the highest swelling capacity, whereas the HSPS-3 showed the lowest water uptake. This behavior is attributed to the reduced mesh size and restricted chain relaxation associated with increasing crosslink density, which limits water diffusion into the hydrogel network. Similar trends have been reported in previous studies [51]. The swelling test in water (Figure 9A), the degree of swelling was higher for the lower crosslinking agent concentration samples. The sharp self-expansion of PAM only in distilled water (Figure 9A), compared to the one obtained in saline solutions of 0.9 wt% NaCl and CaCl_2_ (Figure 9B,C) may result from osmotic pressure differences between the hydrogel interior and external solution. Upon immersion of the hydrogel in distilled water, a large osmotic gradient is formed within the network, and the driving force toward external water entering the PAM matrix increases, accompanied by full polymer swelling. The effect is even greater owing to polar amide (–CONH_2_) groups able to easily bind water molecules. Conversely, in the presence of Na^+^-, Ca^+^- and Cl^−^-ions from the salt solution, osmotic pressure differences between the polymer network and its surroundings decrease. Such ions act as an efficient screen to hydrophilic groups belonging to PAM, thereby impairing water uptake into the gel and hence causing decreased swelling. Moreover, competition of Na^+^ and Ca^+^ ions with the amide groups for hydrogen bonding would reduce their ability to hydrate them.

WRR of HSPS-1, HSPS-2, and HSPS-3 over a 120 h test period is presented in Figure 9D. The measurements were conducted in distilled water under ambient laboratory conditions (24–25 °C and 25% relative humidity). All hydrogels exhibited a rapid decrease in water retention within the first 24 h, with WRR decreasing from 100% to approximately 13% for HSPS-1 and to about 2% for both HSPS-2 and HSPS-3, followed by gradual stabilization. Among the investigated formulations, HSPS-1 retained slightly more water than the more densely crosslinked samples, indicating that a lower crosslinking density enhances water retention owing to the formation of a looser polymer network structure.

Figure 9E presents the swelling behavior of the HSPSs in different aqueous media as a bar chart. The swelling ratio was highest in distilled water and significantly decreased in 0.9 wt% NaCl and 0.9 wt% CaCl_2_ solutions. The stronger effect of Ca^2+^ ions compared to Na^+^ further demonstrates the influence of multivalent cations on hydrogel network contraction.

Figure 9F shows the results of successive swelling/deswelling cycles for an HSPS-1 sample in deionized water. As shown in the graph, the material exhibits a high swelling 3000% even after several cycles. A gradual decrease is observed, from approximately 3500% in the first cycle to about 3000% by the fifth cycle. This result indicates good polymer stability under repeated water exposure, although a slight decrease in water retention capacity is observed with increasing cycles. The appearance of the synthesized polymer hydrogels is shown in Figure 9G and clearly demonstrates the effect of crosslinking density. The HSPS-1 remains mostly transparent and low-viscosity, while the HSPS-2 sample exhibits moderate turbidity and increased gel consistency. In contrast, the HSPS-3 forms a highly opaque, dense gel due to its restricted polymer network. All swelling and water retention measurements were performed in triplicate (*n* = 3). The reported values are presented as the mean ± standard deviation (SD).

**Scheme 1:**
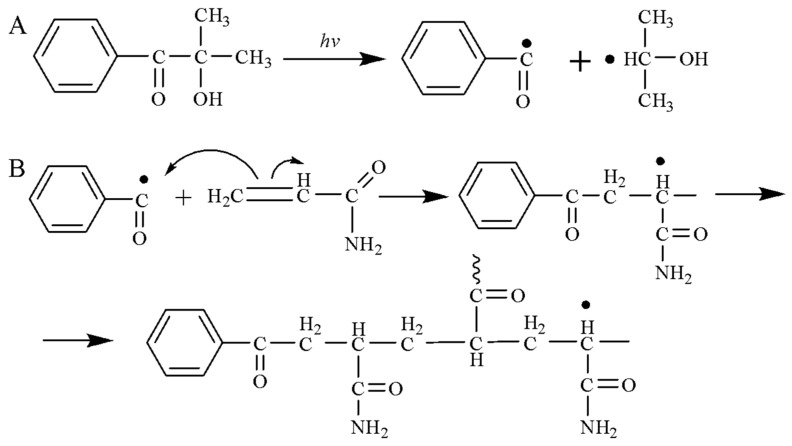
Mechanism of photoinitiator HMP action in the synthesis of PAM: (**A**) formation of two free radicals; (**B**) reaction between benzoyl radical and the monomer.

HMP is a photoinitiator of radical polymerization that is particularly effective under UV irradiation. It belongs to the Type I photoinitiator class, meaning it initiates polymerization via homolytic cleavage, forming free radicals. The mechanism of HMPP photoinitiation can be described as follows (Scheme 1). Upon exposure to UV light, HMPP absorbs energy, transitions to an excited state, and subsequently undergoes cleavage. This photolytic decomposition generates two radicals: a benzoyl radical and an α-hydroxyalkyl radical (Scheme 1A). The benzoyl radical attacks the C=C of the monomer, thereby initiating the chain-growth radical polymerization (Scheme 1B) [52]. The active radical then successively adds acrylamide monomer units, thereby elongating the polymer chain. The reaction proceeds until a crosslinked network is formed.

### 3.3. Swelling Kinetics

**Figure 10:**
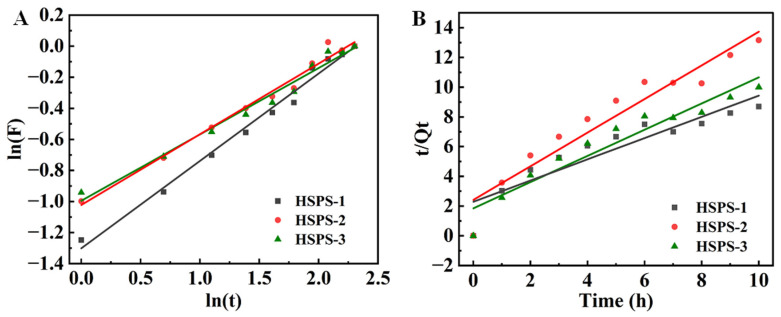
(**A**) Fickian diffusion model; (**B**) Schott’s pseudo-second-order kinetic model.

The swelling behavior of HSPS-1, HSPS-2, and HSPS-3 was investigated by analyzing the swelling kinetics using the Fickian diffusion model and the pseudo-second-order Schott kinetic model. The results are presented in Figure 10A,B. Three theoretical modes can be assumed according to the limit of n: (1) *n* < 0.5, Fickian diffusion is leading, which can be interpreted as the transport power of concentration gradients; (2) 0.5 < *n* < 1, transport is abnormal, because the water adsorption is controlled cooperatively by water diffusion and spread of molecular chains (non-Fickian diffusion); (3) *n* > 1, diffusion system (anomalous diffusion) would be commanded by the stretch and extension of polymer chains [35,53]. In this study, the logarithmic plots of *ln*(*F*) versus *ln*(*t*) showed good linearity for all samples, with diffusion exponent n increasing from 0.89 for the HSPS-1 to 0.95 and 0.97 for the HSPS-2 and HSPS-3, respectively (Figure 10A). These n values indicate an anomalous (non-Fickian) diffusion mechanism, where both water diffusion and polymer chain relaxation contribute to the swelling process. The corresponding *R*^2^ values of 0.983, 0.992, and 0.988 confirm the reliability of the model fitting. The pseudo-second-order kinetic model proposed by Schott assumes that the swelling process is controlled by the availability of free sites within the hydrogel network and by the interaction between water molecules and the polymer chains. In this model, the linearity of the *t*/*Q_t_* versus t plot indicates that the swelling kinetics can be effectively described by this approach, reflecting a process that involves both diffusion and polymer relaxation mechanisms.

Figure 10B presents the plot of *t*/*Q_t_* versus t based on the Schott model, which effectively describes the overall swelling kinetics. In this study, the *t*/*Q_t_* versus t plots demonstrated excellent linearity for all hydrogels, with corresponding *R*^2^ values of 0.997, 0.998, and 0.998 for the HSPS-1, HSPS-2, and HSPS-3 samples, respectively. The calculated equilibrium swelling capacities (*Q_e_*, cal)—13.62 g/g, 8.85 g/g, and 6.20 g/g—were in good agreement with the experimentally determined values (*Q_e_*, exp) of 12.69 g/g, 8.51 g/g, and 5.94 g/g, respectively. It should be noted that these *Q_e_* values were obtained from kinetic model fitting using swelling data collected during the first 10 h of the experiment. The kinetic parameters obtained from both models are summarized in Table 3. Moreover, the observed decrease in the pseudo-second-order rate constant (*K*_2_) with increasing crosslinking degree indicates that higher crosslinking density restricts the polymer network mobility and reduces water uptake rate, in line with the expected trends for highly crosslinked hydrogels.

## 4. Results and Discussion on SWCC Testing

### 4.1. Variations of Soil Suction and Pressure

**Figure 11:**
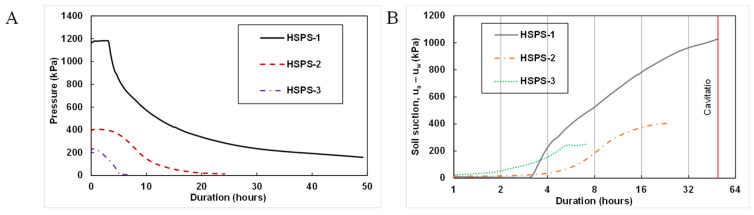
(**A**) Variations of pressure with time and (**B**) variations of soil suction with time.

The HSPS were mixed with different polymers and fixed on the Turan soil to test the capability of pressure and soil suction. The pressure-soil suction relationship was plotted for all the polymers generated here (Figure 11). Significantly, soil suction is plotted in a log scale on Figure 11B to be consistent with the conventional SWCC plots. Monitoring was sustained until the HSPS reached peak pressure (approximately 48 h). The results show that the swelling index (within the group) has a dominant effect on the pressure/suction potential of soil; out of all types, HSPS-3 reflects maximum pressure or suction (pressure = −suction), then HSPS-2 is less than HSPS-1. More swell response, combined with the shape memory of the polymer after re-exposure to water, shows better soil suction.

The measured suction level for each HSPS is indeed very low, and there are two main reasons why the suction force of each HSPS is at such a low level. Cavitation marks the point where all HSPS reach their maximum thresholds, and the liquid phase is turned to air. This effect is shown in the case of HSPS-3 and -2, as these polymers didn’t give a hydrogel in water. In this respect, the remaining HSPSs gelled and reduced the osmotic gradient in the polymer-soil system, providing unsuitable saturation conditions that hinder them from measuring higher suctions.

The same setting time of 24–48 h was observed for equilibrium conditions for all polymers. Most of the HSPSs were stabilised within a day or so, but the induced suction may not have been accurate. After one day, one of the HSPS-3 reaffirmed a level suction at approximately 800 kPa, and all other polymers were between 200 and 400 kPa. Considering the soil types were similar in both samples tested, it can be concluded that HSPS-3 produces the most accurate results under such conditions as water in the extracts being desorbed. Also, HSPS-3 was slower than the others to reach equilibrium and reached a value of about 1020 kPa; higher suctions took longer for equalization. While the soil suction of HSPS-3 was transiently increased at 6 h, the increase decreased by 48 h. Such a decrease is unexpected when the soil was under evaporation conditions that usually lead to greater suction with time. This means that for this polymer, cavitation had already happened at this pressure, and the maximum suction could be calculated to 1 020 kPa.

### 4.2. Temperature Response

**Figure 12:**
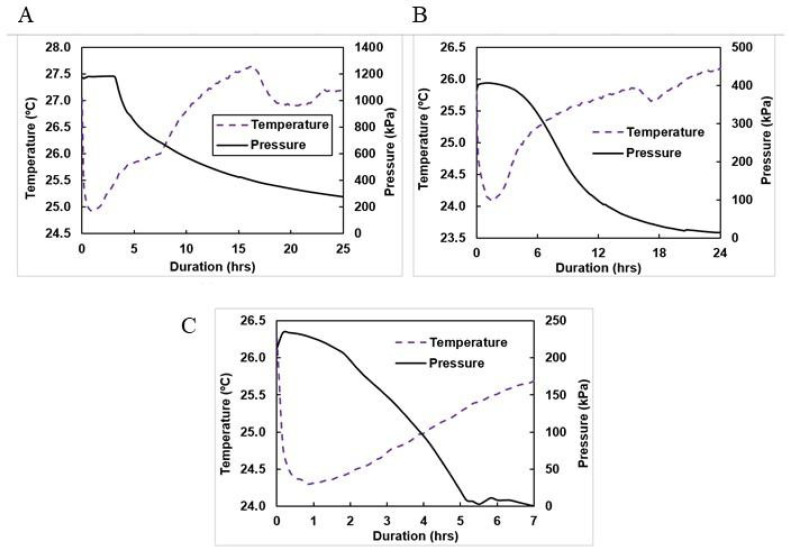
Temperature response of (**A**) HSPS-1, (**B**) HSPS-2, and (**C**) HSPS-3.

The temperature response of all HSPS is shown in Figure 12, indicating the data gathered when monitoring SWCC. The results from this figure show that the pressure generated due to the swelling of the polymer remained stable despite the considerable temperature variation of the sensor’s outer environment, with no active temperature control. The fact that at almost room temperature, the sensor can certainly measure soil suction, confirming that the sensor should carry out SWCC measurement without calibrating for temperature fluctuations. The problem of pressure drop due to temperature results has been greatly researched. Liu et al. [31,32,33] investigated the findings of pressure to rise, concluding that the duration of pressure removal results in the creation of the formed pressure to peak pressure inside a substance in compression rather than the influence of sensor leakage closing up the polymer. One indirect conclusion to the initial pressure decay reduction problem, as explained by the authors, is the minor pressure fluctuation attributed to partial polymer decomposition, which does not compromise the thermal stability confirmed by the polymer TGA analysis. It was deduced in their study by Liu et al. [32] after soil suction measurement. This also corresponds to the report of Lambert [54], explaining that pressure can complicate weight gain due to polymer degradation to monomer. Polymeric degradation occurs in an adhered manner due to hydrogen interaction (H+). The interaction dissolves some compounds, leading to the decreasing meso mass and mechanical modification, which causes alterations to molecular weight. The duration of pressure removal and polymer purity were focused on solving the pressure fade. Pressure relaxation duration is very much under the polymer longevity influence. In case the polymer advances into a hydrogel style, it confines the bio current difference between the sensor and the soil, thus breaking the balance of water, such that the surplus sensor water content won’t go to the core according to the sensor environment. If the balance breaks, there won’t be any pressure relief. However, it does not imply that some salts or ions have been eliminated from the water because the polymer has become full. However, the polymer can greatly swell and suction reading, and eventually, the pressure will degrade when the polymer undergoes some internal degradation. All these were critically studied, and a complete hydration of HSPS was critically considered to make the polymer pure.

As shown in Figure 12A, HSPS-1 shows a relatively stable pressure throughout the monitoring, with a maximum value reached about 1200 kPa, which is then followed by the gradual decrease in response to ambient temperature fluctuations. This observation indicates that within the room temperature range, the 1% PAM formulation is not easily affected by minor temperature changes, which is beneficial for SWCC measurement, where temperature calibration cannot be conducted.

For HSPS-2 and HSPS-3, sharp drops of pressure were observed during the monitoring, attributed to cavitation occurring within the polymer-sensor system. This behavior is indicated by the lower maximum suction capacity compared to the HSPS-1. The more densely crosslinked formulations cause the collapse of the osmotic gradient between the polymer and the soil, which leads to the declining pressure.

For the case of Polyacrylamide, a HSPS-1 perspective will help limit slow pressure relaxation during short-term testing. However, the results seem to indicate that there is no critical pressure degradation on the composite, which in the future will show whether they have long-term strength of the developed matrix. The work of Hamdany et al. [8] purports the few degradations of cross-linked polymers. The data suggest that HSPS-1 is the most efficient for long-term measurement because they have shown strong resistance to the above condition, showing that the relaxation takes a short time.

### 4.3. Soil–Water Characteristics Curves (SWCC)

**Figure 13:**
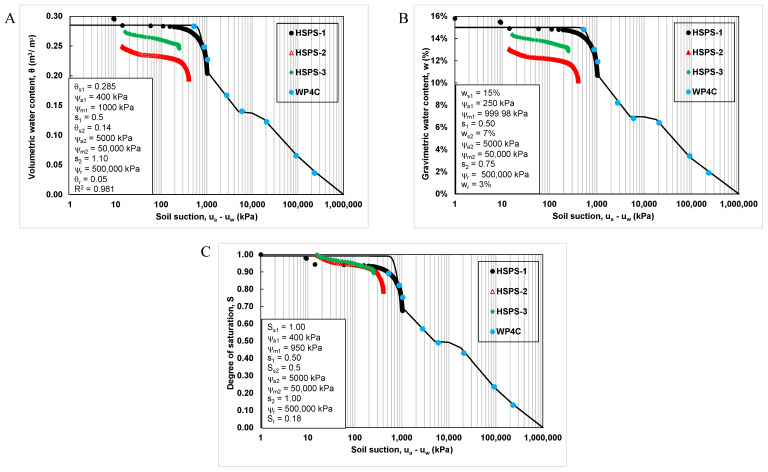
SWCC in terms of (**A**) volumetric water content, (**B**) gravimetric water content, and (**C**) degree of saturation.

The SWCC results of all three polymers are plotted in Figure 13, and the best-fitting line is given only for HSPS-3 to achieve better accuracy relative to other preparations of HSPS. The findings are discussed against the background of soil with bimodal content (coarse and fine-grained fractions). The first sigmoid curve, however, is related to the percentage of coarse material, as indicated by AEV-1 at approximately 400 kPa suction. This phenomenon may be caused by the coarse material, which is mainly composed of medium to fine sand with nearly standardized grain size, as opposed to coarser sand with homogenous pores. Larger particle sizes and linear pore size distribution usually result in lower AEV values, and higher suction rates are satisfied at lower suctions.

By contrast, the second sigmoidal curve is controlled by the fine material content and starts at ~10,000 kPa, which coincides with the top of the high suction range soil measurements done using WP4C. The occurrence of AEV-2 close to the defined height suction point indicates that the soil has a somewhat narrow pore size distribution, for which large energy is required to drain effectively. Also, the remaining suction of 500,000 kPa shows extensive water residual due to a small pore size. Because clay, which corresponds to the fines fraction and is finer than silt particle size 2 μm, is present, it is justified to expect reasonable values of fitted parameters for the second sigmoidal curves in the high-suction domain. It is consistent with previous investigations of Astana soil, with a significant portion of coarse-grained soil. Despite composing a minor portion of the overall particle size distribution, the fine content has an inevitable effect on the soil behavior. Sagidullina et al. [55] confirmed the minor presence of clay (illite) within the fines fraction governing the micropores structure, while the larger portion of sand governs the macropores, which causes the dual pore-size distribution to produce the bimodal SWCC.

There were magnitudes of discrete data points that did not have the best-fit line on SWCC with HSPS-1%. This variability is largely attributed to the measurement system used being a manual weighing scale and not a moisture sensor, which causes penetrometer readings to be made on fluctuating soil mass. As a moisture sensor was not available, a regular scale was used, and the addition of moisture in future studies could likely provide more accurate results. The HSPS uses direct measurements of suction as measured during each time step to examine changes in suction. As such, sample weights need to be recorded at the same regular intervals to avoid analytical mistakes. While there is some dispersion among the points, most of them fall pretty closely along the best-fit line, indicating a consistent overall message.

The comparison results show that the HSPS data given in the S-SWCC section are close to the fitted line by a large degree in comparison with other forms of SWCC. This conjecture is inspired by the results of Leong [56] and Aventian et al. [7], Zhai et al. [57], who noted that the S-SWCC provides a realistic description of volumetric response of the soil during desaturation. When we use the best φ for each data set, our optimal line does not fit other data except for HSPS-3, which has been used to make the EQ “sup” sign (Figure 13B) compelling. However, future investigations are required to verify these outcomes as well as measure the soil volume changes during the entire process of SWCC measurement, such as 3D scanning technology.

Furthermore, only HSPS-1 for all polymers tested adequately represents soil suction from these SWCCs. The result of the unsaturated soil parameter is consistent with that given by Aventian et al. [38] due to the same type of soil used. The first sigmoidal curve differs, as Aventian et al. [38]. In this study, an improvised Tempe Cell was used to verify the accuracy of the SWCC. However, some of the HSPS are not suitable when a polymer forms a hydrogel, and lower strength is achieved, leading to less accurate readings.

The new polymer has great potential for SWCC monitoring, especially HSPS-3, which is very close to the optimal line. This alternative approach of polymer is used to estimate soil suction, which widens the functioning range of the WP4C. Additionally, the apparatus for and process of axis translation are less complex and take less time to accomplish than required preparation times (weeks to months in some cases) or monitoring. Although these new polymers exhibit lower suction strength compared to other experiments, demonstrators can be more easily produced. Liu et al. [31] prepared a polymer through UV light, which could be less available to many of the researchers and places. Compared with HSPS-1 and -2, the fabrication process of HSPS-3 is relatively simpler, which needs less equipment, so that it can more easily be applied to mass production. As a result, the polymer in HSPS-3 represents an alternative to measure matric potentials up to 1 MPa.

Further research should focus on the implementation of HSPS in shear strength testing equipment (modified triaxial) to measure the unsaturated shear strength. Gao et al. [49], the method for calculating unsaturated shear strength would refer to that by Gao et al. [48] and Niu et al. [58]. It is of interest to determine the effect of freezing and the time required for suction equilibration, in addition to the overall time required for these labour-intensive measurements.

## 5. Conclusions

This study demonstrated the feasibility of using UV-synthesized PAM hydrogels as sensing media in an HSPS for soil suction measurement and SWCC determination. The findings highlight that the crosslinking density plays a critical role in governing the swelling behavior, water retention properties, and suction-sensing performance of the synthesized hydrogels. Among the synthesized samples, HSPS-3 showed the best results, providing the highest values of pressure and suction capacity. Furthermore, HSPS-3 is compatible with existing soil suction measurement approaches and can be used as a complementary sensing medium alongside the WP4C instrument. Overall, the developed UV-synthesized polymer system demonstrates considerable potential for the development of alternative and user-friendly technologies for monitoring unsaturated soils. Future work should focus on integrating the HSPS into shear strength testing devices and further optimizing the polymer formulation to improve its accuracy, reliability, and long-term durability. Another recommendation for future work is to evaluate the HSPS considering multiple soils with varying textures and plasticity to assess its performance under different soil conditions.

## Figures and Tables

**Table 1:** Compositions of the syntheses of polymers.

Samples	Degree of Crosslinking: %	Amount of Monomer: g	Amount of Crosslinking: g	Amount of Photoinitiator: mg	The Volume of Solution: mL
HSPS-1	1	2.5	0.025	1	30
HSPS-2	5	2.5	0.125	1	30
HSPS-3	10	2.5	0.25	1	30

**Table 2:** Soil properties.

Soil Properties	Value	Standards
Specific gravity, *G*s	2.50	ASTM-D854-02 [39]
Water content, *w* (%)	2.19	ASTM-D2216-10 [40]
Plastic limit, PL (%)	19.33	ASTM-D4318-00 [41]
Liquid limit, LL (%)	39.58	
Plasticity index, *PI*	20.25	
Gravel (%)	0	ASTM-D422-63 [42]
Sand (%)	87.24	
Fines (%)	12.64	
Unified Soil Classification System (USCS)	Clayey sand (SC)	ASTM-D2487-00 [43]
Dry density at maximum condition, $\gamma_{dmax}$ (gr/cm^3^)	1.91	ASTM-D698-12 [44]
Water content at optimum condition, OMC (%)	13.50	

**Table 3:** Kinetic parameters for the swelling of HSPSs with different crosslinking degrees based on Fickian and Schott models.

Samples	*n*	*R*^2^ (Fick)	*Q_e_*_exp (g/g)	*Q_e_*_cal (g/g)	*K*_2_ × 10^5^ (g^−1^·min^−1^)	*R*^2^ (Schott)
HSPS-1	0.89	0.983	12.69	13.62	2.36	0.997
HSPS-2	0.95	0.992	8.51	8.85	1.89	0.998
HSPS-3	0.97	0.988	5.94	6.20	1.57	0.998

## Data Availability

The original contributions presented in this study are included in the article. Further inquiries can be directed to the corresponding author.

## References

[1] Ahmed E.M. (2015). Hydrogel: Preparation, characterization, and applications: A review. J. Adv. Res..

[2] Chavan V.D., Pinjari D.V., Waghmare N.G., Juikar V.C., Sayyed A.J. (2024). A critical review on technological development of cellulosic material as a sustainable alternative for superabsorbent polymers and its recent applications. Chem. Eng. J..

[3] Mignon A., De Belie N., Dubruel P., Van Vlierberghe S. (2019). Superabsorbent polymers: A review on the characteristics and applications of synthetic, polysaccharide-based, semi-synthetic and ‘smart’ derivatives. Eur. Polym. J..

[4] Jamali F., Etminani-Esfahani N., Rahmati A. (2023). Maleic acid is an important monomer in synthesis of stimuli-responsive poly(acrylic acid-co-acrylamide-co-maleic acid) superabsorbent polymer. Sci. Rep..

[5] Chen Y.-C., Chen Y.-H. (2019). Thermo and pH-responsive methylcellulose and hydroxypropyl methylcellulose hydrogels containing K_2_SO_4_ for water retention and a controlled-release water-soluble fertilizer. Sci. Total Environ..

[6] Poy A., Maity P.P., Bose A., Dhara S., Pal S. (2019). β-Cyclodextrin based pH and thermo-responsive biopolymeric hydrogel as a dual drug carrier. Mater. Chem. Front..

[7] Aventian G.D., Satyanaga A., Arinova A., Kalimuldina G., Moon S.-W., Kim J. (2025). Variability of polymer for determination of soil-water characteristic curves. J. Rock Mech. Geotech. Eng..

[8] Hamdany A.H., Shen Y., Satyanaga A., Rahardjo H., Lee T.T.D., Nong X. (2022). Field instrumentation for real-time measurement of soil-water characteristic curve. Int. Soil Water Conserv. Res..

[9] Dalida M.L.P., Mariano F.V., Futalan C.M., Kan C.-C., Tsai W.-C., Wan M.-W. (2011). Adsorptive removal of Cu(II) from aqueous solutions using non-crosslinked and crosslinked chitosan-coated bentonite beads. Desalination.

[10] Kristo C., Rahardjo H., Satyanaga A. (2017). Effect of variations in rainfall intensity on slope stability in Singapore. Int. Soil Water Conserv. Res..

[11] Wang X., Cui J., Zhou J., Wang S., Gu Y., Liu X. (2023). Preparation of polyacrylamide hydrophilic stationary phases with adjustable performance. Chromatogr. A.

[12] Liu Y., Zheng H., Si B., Zheng X. (2018). Optimized synthesis of polyacrylamide (PAM) with UV/H2O2 initiating system and evaluation of its application performance. Desalin. Water Treat..

[13] Li Y., Satyanaga A., Rahardjo H. (2021). Characteristics of unsaturated soil slope covered with capillary barrier system and deep-rooted grass under different rainfall patterns. Int. Soil Water Conserv. Res..

[14] Or D. (2001). Who Invented the Tensiometer?. Soil Sci. Soc. Am. J..

[15] Ridley A.M., Burland J. (1993). BA new instrument for the measurement of soil moisture suction. Geotechnique.

[16] Toll D.G., Lourenço S.D.N., Mendes J. (2013). Advances in suction measurements using high suction tensiometers. Eng. Geol..

[17] Mendes J., Gallipoli D., Boeck F., Tarantino A. (2020). Comparative study of high capacity tensiometer designs. Phys. Chem. Earth Parts A/B/C.

[18] Mendes J., Jamali A., Najdi A., Encalada D., Bruno A.W., Prat P.C., Buzzi O., Gallipoli D., Ledesma A., Toll D. High Capacity Tensiometers: Performance and Behaviours. Proceedings of the 8th International Conference on Unsaturated Soils (UNSAT 2023).

[19] Satyanaga A., Rahardjo H., Koh Z.H., Mohamed H. (2019). Measurement of a soil-water characteristic curve and unsaturated permeability using the evaporation method and the chilled-mirror method. J. Zhejiang Univ. Sci. A.

[20] Kristo C., Rahardjo H., Satyanaga A. (2019). Effect of hysteresis on the stability of residual soil slope. Int. Soil Water Conserv. Res..

[21] Lagerwerff J.V., Ogata G., Eagle H.E. (1961). Control of Osmotic Pressure of Culture Solutions with Polyethylene Glycol. Science.

[22] Peck A.J., Rabbidge R.M. (1966). Soil-water potential: Direct measurement by a new technique. Science.

[23] Peck A.J., Rabbidge R.M. (1969). Design and Performance of an Osmotic Tensiometer for Measuring Capillary Potential. Soil Sci. Soc. Am. J..

[24] Kassiff G., Shalom A.B. (1971). Experimental Relationship Between Swell Pressure and Suction. Géotechnique.

[25] Bocking K., Fredlund D. (1979). Use of the Osmotic Tensiometer to Measure Negative Pore Water Pressure. Geotech. Test..

[26] Biesheuvel P.M., Raangs R., Verweij H. (1999). Response of the Osmotic Tensiometer to Varying Temperatures Modeling and Experimental Validation. Soil Sci. Soc. Am. J..

[27] Biesheuvel P.M., Van Loon A.P., Raangs R., Verweij H., Dirksen C. (2000). A prototype osmotic tensiometer with polymeric gel grains. Eur. J. Soil Sci..

[28] Bakker G., van der Ploeg M.J., de Rooij G.H., Hoogendam C.W., Gooren H.P.A., Huiskes C., Koopal L.K., Kruidhof H. (2007). New Polymer Tensiometers: Measuring Matric Pressures Down to the Wilting Point. Vadose Zone.

[29] van der Ploeg M.J. (2008). Polymer Tensiometers to Characterize Unsaturated Zone Processes in Dry Soils. Ph.D. Thesis.

[30] van der Ploeg M.J., Gooren H.P.A., Bakker G., Hoogendam C.W., Huiskes C., Koopal L.K., Kruidhof H., de Rooij G.H. (2010). Polymer tensiometers with ceramic cones: Direct observations of matric pressures in drying soils. Hydrol. Earth Syst. Sci..

[31] Liu H., Rahardjo H., Satyanaga A., Du H. (2021). Use of synthesised polymers for the development of new osmotic tensiometers. Geotechnique.

[32] Liu H., Rahardjo H., Du H., Hamdany A.H. (2023). Long-Term Decay of the Water Pressure in the Osmotic Tensiometer. J. Rock Mech. Geotech. Eng..

[33] Liu H., Rahardjo H., Satyanaga A., Du H. (2023). Use of Osmotic Tensiometers in the Determination of Soil–Water Characteristic Curves. Eng. Geol..

[34] He M., Ou F., Wu Y., Sun X., Chen X., Li H., Sun D., Zhang L. (2020). Smart Multi-Layer PVA Foam/CMC Mesh Dressing with Integrated Multi-Functions for Wound Management and Infection Monitoring. Mater. Des..

[35] Chen M., Ni Z., Shen Y., Xiang G., Xu L. (2020). Reinforced swelling and water-retention properties of super-absorbent hydrogel fabricated by a dual stretchable single network tactic. Colloids Surf. A Physicochem. Eng. Asp..

[36] KELLER (2022). Data Sheet Series 35X Pressure Transmitter.

[37] Aventian G.D., Satyanaga A., Sagu A., Serikbek B., Pernebekova G., Aubakirova B., Zhai Q., Kim J. (2024). Analytical and Finite-Element-Method-Based Analyses of Pile Shaft Capacity Subjected to Rainfall Infiltration. Sustainability.

[38] Aventian G.D., Satyanaga A., Zhakiyeva A., Hamdany A.H., Wijaya M., Irawan S., Kim J. (2024). High-Suction Polymer Sensor for Measurement of Soil Suction under Freezing and Thawing Conditions. Cold Reg. Sci. Technol..

[39] (2002). Standard Test Methods for Specific Gravity of Soil Solids by Water Pycnometer.

[40] (2010). Test Methods for Laboratory Determination of Water (Moisture) Content of Soil and Rock by Mass.

[41] (2000). Standard Test Methods for Liquid Limit, Plastic Limit, and Plasticity Index of Soils.

[42] (2007). Standard Test Method for Particle-Size Analysis of Soils.

[43] (2000). Standard Classification of Soils for Engineering Purposes (Unified Soil Classification System).

[44] (2012). Standard Test Methods for Laboratory Compaction Characteristics of Soil Using Standard Effort (12 400 ft-lbf/ft3 (600 kN-m/m3)).

[45] Guan G.S., Rahardjo H., Choon L.E. (2009). Shear Strength Equations for Unsaturated Soil under Drying and Wetting. J. Geotech. Geoenviron. Eng..

[46] Li B., Liu J., Fu D., Li Y., Xu X., Cheng M. (2021). Rapid preparation of PAM/N-CNT nanocomposite hydrogels by DEM frontal polymerization and its performance study. RSC Adv..

[47] Wang X.N., Yue Q.Y., Gao B.Y., Si X.H., Sun X., Zhang S.X. (2011). Dispersion copolymerization of acrylamide and dimethyl diallyl ammonium chloride in ethanol-water solution. J. Appl. Polym. Sci..

[48] Sun Y.J., Zhu C.Y., Sun W.Q., Xu Y.H., Xiao X.F., Zheng H.L., Wu H.F., Liu C.Y. (2017). Plasma-initiated polymerization of chitosan-based CS-g-P(AM-DMDAAC) flocculant for the enhanced flocculation of low-algal-turbidity water. Carbohyd. Polym..

[49] Gao Y., Sun D., Zhu Z., Xu Y. (2019). Hydromechanical behavior of unsaturated soil with different initial densities over a wide suction range. Acta. Geotech..

[50] Kumar M.A., Moghal A.A.B., Rasheed R.M., Rehman A.U. (2025). Enhancing Durability and Erosion Resistance of Soils with Varying Plasticity Using Crosslinked Biopolymers. Sci. Rep..

[51] Pal P., Pandey J.P., Sen G. (2018). Chapter 5-Synthesis and application as programmable water soluble adhesive of polyacrylamide grafted gum tragacanth (GT-g-PAM). Biopolym. Grafting.

[52] Decker C. (1996). Photoinitiated crosslinking polymerization. Prog. Polym. Sci..

[53] Huang Y., Zhang B., Xu G., Hao W. (2013). Swelling behaviours and mechanical properties of silk fibroin–polyurethane composite hydrogels. Compos. Sci. Technol..

[54] Lambert S. (2013). Environmental Risk of Polymers and Their Degradation Products. Ph.D. Thesis.

[55] Sagidullina N., Satyanaga A., Kim J., Moon S.-W. (2025). Engineering Behavior and Geotechnical Challenges of Sulfate-Rich Soils in Astana. Front. Built Environ..

[56] Leong E.-C. (2019). Soil-water Characteristic Curves—Determination, Estimation and Application. Jpn. Geotech. Soc. Spec. Publ..

[57] Zhai Q., Rahardjo H., Satyanaga A., Dai G., Zhuang Y. (2020). Framework to estimate the soil-water characteristic curve for soils with different void ratios. Bull. Eng. Geol. Environ..

[58] Niu G., Sun D., Kong L., Shao L., Wang H., Wang Z. (2024). Investigation into the shear strength of a weakly expansive soil over a wide suction range. Acta. Geotech..

